# Improved MRD 4H-SiC MESFET with High Power Added Efficiency

**DOI:** 10.3390/mi10070479

**Published:** 2019-07-17

**Authors:** Shunwei Zhu, Hujun Jia, Xingyu Wang, Yuan Liang, Yibo Tong, Tao Li, Yintang Yang

**Affiliations:** School of Microelectronics, Xidian University, Xi’an 710071, China

**Keywords:** 4H-SiC, MESFET, IMRD structure, power added efficiency

## Abstract

An improved multi-recessed double-recessed p-buffer layer 4H–SiC metal semiconductor field effect transistor (IMRD 4H-SiC MESFET) with high power added efficiency is proposed and studied by co-simulation of advanced design system (ADS) and technology computer aided design (TCAD) Sentaurus software in this paper. Based on multi-recessed double-recessed p-buffer layer 4H–SiC metal semiconductor field effect transistor (MRD 4H-SiC MESFET), the recessed area of MRD MESFET on both sides of the gate is optimized, the direct current (DC), radio frequency (RF) parameters and efficiency of the device is balanced, and the IMRD MESFET with a best power-added efficiency (PAE) is finally obtained. The results show that the PAE of the IMRD MESFET is 68.33%, which is 28.66% higher than the MRD MESFET, and DC and RF performance have not dropped significantly. Compared with the MRD MESFET, the IMRD MESFET has a broader prospect in the field of microwave radio frequency.

## 1. Introduction

Nowadays, as the device size continues to decrease, the process difficulty increases significantly, both the power consumption and non-ideal effects are significant. The first-generation semiconductor Si and other materials are close to their theoretical limits in performance, while 4H Silicon Carbide (4H-SiC) has a wide band gap (3.26 eV), high thermal conductivity (4.9 W/(cm·K)), high breakdown electric field (4 MV/cm) and low dielectric constant (9.7), high electron saturation drift speed (2.7 × 10^7^ cm/s), and exhibits superior performance compared to 3C-SiC, 6H-SiC, Si, GaAs, and so on. Based on the excellent characteristics of 4H-SiC, 4H-SiC metal semiconductor field transistors (4H-SiC MESFETs) are expected to be applied to various semiconductor fields [1,2,3]. However, current research on 4H-SiC MESFET mainly includes breakdown voltage, saturation drain current, electron saturation drift speed, frequency characteristics etc. [4,5,6,7,8,9,10]. There are a variety of ways to improve device performance, such as the use of double-recessed gates [4], recessed buffers and diffusion regions [5,6], doping distribution modification [7,8], silicon-on-insulator (SOI) technology [9,10] and so on, which can significantly improve device performance. At the same time, there is little research on efficiency [11,12]. For non-conventional 4H-SiC-based FETs, the characteristics of the device can be studied using numerical simulations [13,14,15,16,17].

In this paper, we reported a 4H-SiC MESFET with improved multi-recessed double-recessed p-buffer layer (IMRD) structure. Traditional 4H-SiC MESFETs have been experimentally verified. Many experimental results have been reported so far [18,19], but they are all fixed structures, and the effect of structural parameters on the results has not been studied. Based on multi-recessed 4H–SiC MESFETs with double-recessed p-buffer layer (MRD 4H-SiC MESFET) [20], we adopt a new design method optimized by technology computer aided design (TCAD) simulation and verified in advanced design system (ADS) software. The IMRD MESFET has both the excellent performance of MRD MESFET and better power-added efficiency (PAE) and provides a new idea for high-power operational amplifier design at the device level.

## 2. Device Structure and Description

**Figure 1:**
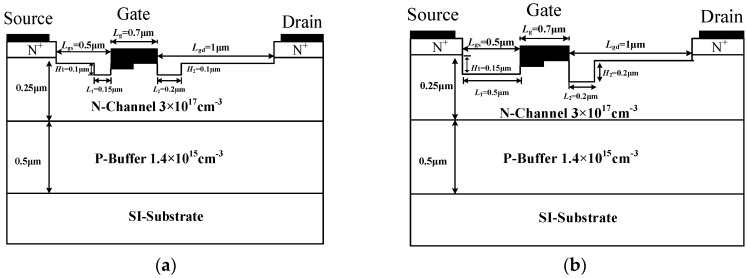
Schematic cross sections of the (**a**) MRD 4H-SiC metal semiconductor field effect transistor (MESFET), (**b**) improved multi-recessed double-recessed p-buffer layer (IMRD) 4H-SiC MESFET.

Figure 1a,b are the cross-sectional views of the MRD MESFET and the IMRD MESFET, respectively. In Figure 1a, the MRD MESFET contains a high-purity semi-insulating substrate (SI-Substrate), a p-type buffer layer (P-Buffer), an n-type channel layer (N-Channel), and two doped n-type cap layers (Source and Drain), the Nickel Schottky gate has a work function of 5.1 eV. By high energy ion implantation and high temperature annealing processes, two recessed areas are formed on both sides of the gate. The main difference between the two devices is the recessed regions on both sides of the gate. The length and width on both sides are *L*_1_ = 0.15 μm, *L*_2_ = 0.2 μm, *H*_1_ = 0.1 μm and *H*_2_ = 0.1 μm, other parameters are shown in Table 1. In the optimized IMRD MESFET, *L*_1_ = 0.5 μm, *L*_2_ = 0.2 μm, *H*_1_ = 0.15 μm and *H*_2_ = 0.2 μm, and the other parameters are consistent with the MRD MESFET.

**Figure 2:**
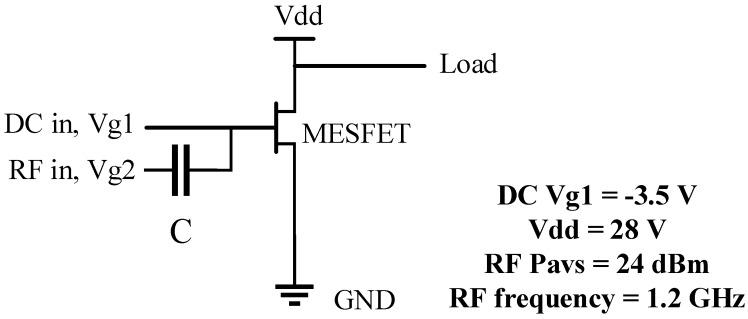
One Tone Load Pull Schematic for power-added efficiency (PAE) measurements.

The 2D TCAD simulator, Sentaurus is used in this paper. The simulation temperature is set to 300 K. A 5.1 eV Nickel Schottky gate work function is applied. The main model used in the simulation are Mobility (Enormal Doping Dep HighFieldsaturation (GradQuasiFermi)), Recombination (Auger SRH (DopingDep)), Incomplete Ionization, Effective Intrinsic and Density (BandGapNarrowing (OldSlotboom)) [21]. The interface state has a great impact on the device, in this paper, the gate of the two devices are formed by a metal-to-SiC contact to form a Schottky contact, so the gate does not have to consider the interface state. When the MESFET is passivated with a material such as Si_3_N_4_ or SiO_2_, the performance is slightly degraded. When verifying the conventional 4H-SiC MESFET, Si_3_N_4_ was used as the passivation layer. When Si_3_N_4_ is used as the passivation layer, the device performance is reduced by less than SiO_2_ [22,23]. As a theoretical analysis, the influence of passivation on the device is not considered here. After obtaining the simulation results, the obtained model parameters are used in the ADS software to measure the PAE of the two devices. In the ADS simulation, the bias conditions of the device are shown in Figure 2. Direct current (DC) voltage Vg1 is −3.5 V, Vdd is 28 V, input power Pavs is 24 dBm, and frequency *f* is 1.2 GHz.

## 3. Results and Discussion

### 3.1. Effect of the Length and Height of the Recessed Regions on the PAE

**Figure 3:**
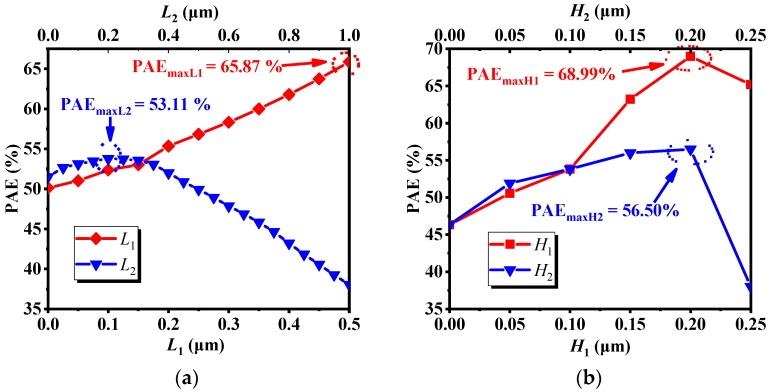
The effect of the (**a**) length and (**b**) height on PAE.

The effect of the length and height of the recessed regions on PAE is shown in Figure 3. When a parameter changes, the remaining parameters are the default values in Figure 1a. We can see in Figure 3a, when *L*_1_ increases, PAE also increases. When *L*_1_ reaches 0.5 μm, PAE reaches a maximum value; when *L*_2_ is less than 0.1 μm, the PAE increases with *L*_2_. When *L*_2_ reaches 0.2 um, PAE reaches a maximum value. When *L*_2_ is larger than 0.1 μm, PAE decreases with the increase of *L*_2_. In Figure 3b, the trend of the effect of *H*_1_ and *H*_2_ on PAE agrees well. When *H*_1_ and *H*_2_ are less than 0.2 μm, PAE of *H*_1_ and *H*_2_ increase with the increase of *H*_1_ and *H*_2_. When *H*_1_ and *H*_2_ reach 0.2 μm, PAE of *H*_1_ and *H*_2_ reach the maximum value. When *H*_1_ and *H*_2_ are larger than 0.2 μm, PAE of *H*_1_ and *H*_2_ decrease as *H*_1_ and *H*_2_ increases.

### 3.2. Optimized Results and Mechanism Discussion

Through further analysis of the results obtained in 3.1, we found that between *L*_1_, *L*_2_, *H*_1_ and *H*_2_ there is a relatively independent relationship. When *L*_2_, *H*_1_ and *H*_2_ take different values, the trend of PAE with *L*_1_ is almost the same as Figure 3a. The maximum value of PAE is found in *L*_1_ = 0.5 μm. In addition, for *L*_2_, *H*_1_ and *H*_2_, the maximum value of PAE are found in *L*_2_ = 0.2 μm, *H*_1_ = 0.2 μm and *H*_2_ = 0.2 μm. Based on the above results, *L*_1_ = 0.5 μm, *L*_2_ = 0.2 μm, *H*_1_ = 0.2 μm and *H*_2_ = 0.2 μm were selected as the optimal structural parameters, and the optimized device was obtained. The main parameters of the device are shown in Table 2. It can be seen from the table that although its saturated drain current *I*_dsat_ is too small, which is 35.2% lower than that of the MRD MESFET, and the DC characteristics are greatly weakened, its PAE reaches 70.85%, which is 33.40% higher than that of the MRD MESFET. 

**Figure 4:**
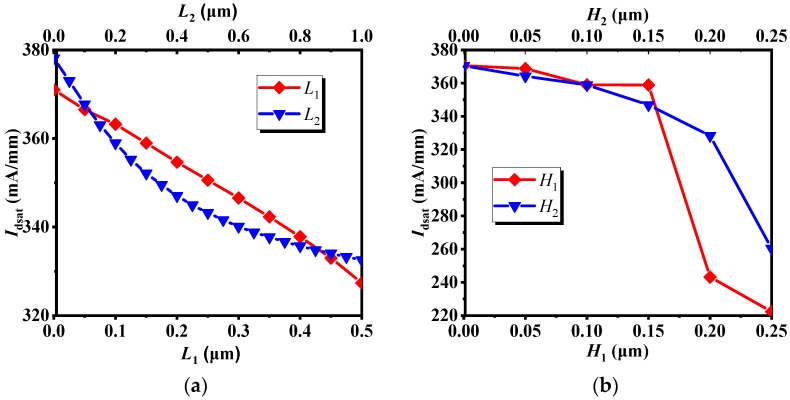
The effect of the (**a**) length and (**b**) height on *I*_dsat_.

Figure 4 shows the effect of the length and height on *I*_dsat_. It can be seen from Figure 4a that when *L*_1_ = 0.5 μm and *L*_2_ = 0.2 μm, the decline of *I*_dsat_ is small; In Figure 4b, it can be clearly seen that when *H*_1_ is less than 0.15 μm, *I*_dsat_ decreases, but the value of decrease is not very large. When *H*_1_ is greater than 0.15 μm, *I*_dsat_ drops sharply. Similarly, for *H*_2_, when *H*_2_ is less than 0.2 μm, the decrease of *I*_dsat_ is not large. When *H*_2_ is larger than 0.2 μm, the decline of *I*_dsat_ is more obvious. The main cause of the weakening of the DC characteristics is that the thickness of the channel region becomes extremely thin, resulting in narrowing the channel of most electrons, and thus the DC characteristics are deteriorated. Based on the results above, *L*_1_ = 0.5 μm, *L*_2_ = 0.2 μm, *H*_1_ = 0.15 μm and *H*_2_ = 0.2 μm were selected as structural parameters, and the PAE was measured to be 68.33%. After optimization, the PAE of the IMRD MESFET is 28.66% higher than that of the MRD MESFET.

**Figure 5:**
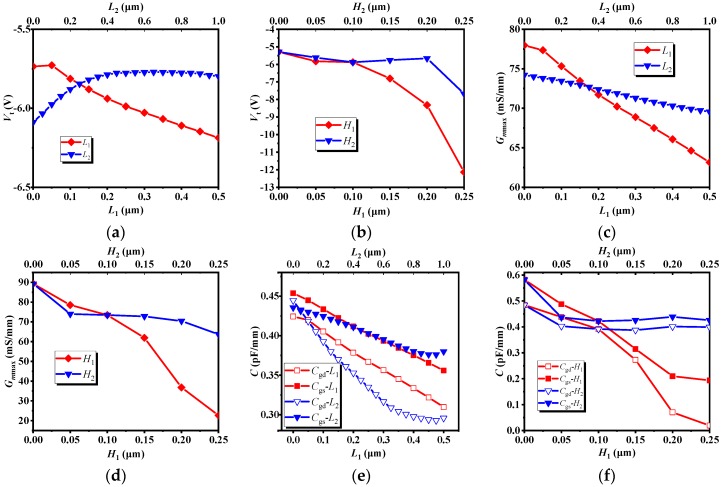
The effect of recessed region parameters on *V*_t_, *Gm*_max_ and *C*_gs_. (**a**) *V*_t_-*L*. (**b**) *V*_t_-*H*. (**c**) *Gm*_max_-*L*. (**d**) *Gm*_max_-*H*. (**e**) *C*-*L*. (**f**) *C*-*H*.

Figure 5 illustrates the effect of recessed region parameters (*L* & *H*) on *V*_t_, *Gm*_max_, *C*_gs_ and *C*_gd_, the transconductance *Gm*_max_, its physical meaning is the first-order partial conductance of the output drain current and the input voltage, *C*_gs_ is the gate-source capacitance, and *C*_gd_ is the gate-drain capacitance. In combination with Figure 3, it can be seen from Figure 5a,c,e that as *L*_1_ increases, the threshold voltage *V*_t_, the transconductance *Gm*_max_, the gate-source capacitance *C*_gs_ and the gate-drain capacitance *C*_gd_ decrease, but the PAE gradually increases; when *L*_2_ increases, *V*_t_ increases first, then tends to be stationary, *Gm*_max_ gradually decreases, the change trend of *C*_gd_ and *C*_gs_ are about the same. When *L*_2_ is less than 0.1 μm, the changes of *C*_gs_ and *C*_gd_ are relatively stable. When *L*_2_ is larger than 0.1 μm, *C*_gs_ and *C*_gd_ are gradually increased. Combined with Figure 3a and Figure 5a,c,e, it can be found that the effects of *C*_gs_ and *C*_gd_ on PAE are obvious. It can be seen from the bias conditions of Figure 2 that *C*_gs_ and *C*_gd_ affect the input impedance and output impedance respectively, and the power consumed by the capacitor is proportional to the size of the capacitor. According to the definition of PAE, these two capacitors affect the input power and output power, so these two capacitors have a greater impact on the PAE. Similarly, in conjunction with Figure 3, it can be seen from Figure 5b,d,f that, when *H*_1_ is less than 0.2 μm, as *H*_1_ increases, *V*_t_, *Gm*_max_, *C*_gs_ and *C*_gd_ decrease, while PAE increases. When *H*_1_ is larger than 0.2 μm, PAE decreases with *H*_1_. For *H*_2_, when *H*_2_ increases gradually, *V*_t_ and *Gm*_max_ also decrease gradually, but when *H*_2_ is less than 0.05 μm, *C*_gs_ and *C*_gd_ decrease with increasing *H*_2_. When *H*_2_ is greater than 0.05 μm, the change of *C*_gs_ and *C*_gd_ are relatively stable. When *H*_2_ is less than 0.2 μm, PAE increases with the increase of *H*_2_. When *H*_2_ is greater than 0.2 μm, PAE decreases as *H*_2_ increases. Combined with Figure 3b, Figure 4b and Figure 5b,d,f, although the capacitances *C*_gs_ and *C*_gd_ have a greater influence on PAE, as the *H*_2_ increases, the conductive channel of the device still decreases. When the thickness of the conductive channel is less than 0.05 μm, the conductive channel is extremely thin, and its conductive properties are greatly weakened. At this time, both the AC signal and the DC signal will be greatly attenuated in the channel, resulting in a decrease in output power. Under the combined action of several factors, the PAE decreases as *H*_2_ increases.

The power-added efficiency (PAE) is the difference in output and input power in place of radio frequency (RF) output power in the drain efficiency equation, which takes power gain *G*_p_ into account.

(1) $$\mathrm{PAE}=\frac{P_{\mathrm{out}}-P_{\mathrm{in}}}{P_{\mathrm{dc}}}=\eta_{c}(1-\frac{1}{G_{p}})$$

*η*_c_ is the drain efficiency and represents the ratio of output power to DC input power. In order to obtain a higher PAE, a larger power gain *G*_p_ is required. It can be seen from the equivalent circuit diagram of the device, the size of the PAE is determined by these parameters together. For the threshold voltage *V*_t_, only when the absolute value of *V*_t_ is reduced, can small voltage make the channel turn on. When the input voltage is constant, the smaller the absolute value of the threshold voltage is, the larger the channel current is, and the larger the output power *P*_out_ is, so that the PAE becomes larger. When *Gm*_max_ is reduced, its DC characteristic will also decrease. Since the input voltage is constant, the decrease of *Gm*_max_ causes the bias current *I*_d_ to decrease, so that the *P*_dc_ is reduced, according to the definition of PAE, the decrease of *P*_dc_ will cause the increase of PAE. The gate-source capacitance *C*_gs_ and the gate-drain capacitance *C*_gd_ have a greater influence on the PAE, in the equivalent circuit, *C*_gs_ and *C*_gd_ are in the input loop and output loop respectively. Because of the larger *C*_gs_ in the bias, the more AC input energy it consumes, the larger the *C*_gd_, the smaller the AC output power of the load is. In addition, as the channel becomes thinner and thinner, it affects both the capacitance and the carrier’s passage through the channel region. Although the PAE increases as the capacitance decreases, the extremely thin channel also makes the DC and AC characteristics affected seriously. From the analysis of 3.1–3.3, when the structural parameters of the device are changed, in order to obtain the optimal PAE, it is necessary to weigh the various parameters and find the optimal structural parameters on the premise that the other performance is guaranteed. Perhaps the above design process will sacrifice a small part of the performance, but the efficiency is greatly improved, achieving energy saving and emission reduction, which is very beneficial to the construction of green earth.

## 4. Conclusions

An improved MRD 4H-SiC MESFET with high power added efficiency is analyzed and studied by co-simulation of ADS and TCAD Sentaurus software in this paper. Based on MRD 4H-SiC MESFET, we optimize the MRD MESFET on both sides of the gate. In the recessed area, the DC, RF parameters and efficiency of the device are weighed, and the IMRD MESFET with the best PAE is finally obtained. The results show that the saturation drain current Idsat of the IMRD MESFET is 311 mA, the threshold voltage *V*_t_ is −6.99 V, the maximum transconductance *Gm*_max_ is 46.37 mS, the gate-source capacitance *C*_gs_ is 0.218 pF, and the gate-drain capacitance *C*_gd_ is 17.9 fF. The PAE is 68.33%, which is 28.66% higher than the MRD MESFET, and DC and RF performance have not dropped significantly. This paper proposes to lay a device-level theoretical basis and design method for further energy-efficient RF power amplifier.

## Figures and Tables

**Table 1:** Common parameters of the two structures.

Parameters	Values
P-Buffer Concentration	1.4 × 10^15^ cm^−3^
N-Channel Concentration	3 × 10^17^ cm^−3^
N-Cap layers Concentration	2 × 10^19^ cm^−3^
*L*_gs_	0.5 μm
*L*_gd_	1.0 μm
*L*_s_	0.5 μm
*L*_d_	0.5 μm
*L*_g_	0.7 μm
N-Channel Thickness	0.25 μm
P-Buffer Thickness	0.5 μm
Device Area (without SI-Substrate)	1 μm × 3.5 μm

**Table 2:** Comparison of performance parameters of the two structures.

Parameters	MRD MESFET	IMRD MESFET
*I*_dsat_ (mA/mm)	358.97	233.02
*g*_m_ (mS/mm)	73.45	56.37
*V*_t_ (V)	−5.81	−6.89
*C*_gs_ (pF/mm)	0.128	0.13
*C*_gd_ (pF/mm)	0.39	0.02
Power-added efficiency (PAE) (%)	53.11	70.85
